# Patient and Citizen Participation in the Identification of Ethical Considerations Aiming to Address Uncertainty in the Evaluation of Promising Interventions in a Pandemic Context

**DOI:** 10.3389/fmedt.2021.794003

**Published:** 2021-12-24

**Authors:** Catherine Olivier, Isabelle Ganache, Olivier Demers-Payette, Louis Lochhead, Sandra Pelaez, Michèle de Guise, Marie-Pascale Pomey

**Affiliations:** ^1^Institut national d'excellence en santé et services sociaux, Bureau - Méthodologies et éthique, Montréal, QC, Canada; ^2^École de santé publique, Université de Montréal, Montréal, QC, Canada; ^3^Centre d'excellence sur le partenariat avec les patients et le public, Montréal, QC, Canada; ^4^École de kinésiologie et des sciences de l'activité physique, Université de Montréal, Montréal, QC, Canada; ^5^Centre de recherche du Centre Hospitalier, Université de Montréal, Montréal, QC, Canada

**Keywords:** patient participation, citizen participation, promising interventions, uncertainty, pandemic, COVID-19, benefit-risk assessment

## Abstract

Since the beginning of the COVID-19 pandemic, numerous studies have been conducted to identify interventions that could contribute to alleviating the burden it has caused. The Institut national d'excellence en santé et en services sociaux (INESSS) has played a key role in informing the government of Québec regarding the evaluation of specific pandemic-related interventions. This process took place in a context characterized by a sense of urgency to assess and recommend potential interventions that could save lives and reduce the effects of the disease on populations and healthcare systems, which increased the pressure on the regulatory agencies leading these evaluations. While some of the interventions examined were considered promising, results from COVID-19 studies often led to uncertainty regarding their efficacy or safety. Regulatory agencies evaluating the value of promising interventions thus face challenges in deciding whether these should be made available to the population, particularly when assessing their benefit-risk balance. To shed light on these challenges, we identified underlying ethical considerations that can influence such an assessment. A rapid literature review was conducted in February 2021, to identify the main challenges associated with the benefit-risk balance assessment of promising interventions. To reinforce our understanding of the underlying ethical considerations, we initiated a discussion among various social actors involved in critical thinking surrounding the evaluation of promising interventions, including ethicists, clinicians and researchers involved in clinical or public health practice, as well as patients and citizens. This discussion allowed us to create a space for exchange and mutual understanding among these various actors who contributed equally to the identification of ethical considerations. The knowledge and perspectives stemming from the scientific literature and those consulted were integrated in a common reflection on these ethical considerations. This allowed patients and citizens, directly affected by the evaluation of pandemic-related interventions and the resulting social choices, to contribute to the identification of the relevant ethical considerations. It also allowed for reflection on the responsibilities of the various actors involved in the development, evaluation, and distribution of promising interventions in a setting of urgency and uncertainty, such as that brought about by the COVID-19 pandemic.

## Introduction

The COVID-19 pandemic has seen an unprecedented mobilization of the scientific community and unparalleled efforts to develop interventions for reducing or countering its impact on individuals and on healthcare systems. These efforts have led to numerous scientific publications aiming to inform regulatory bodies and agencies in their assessments of promising interventions. The body of published scientific evidence often raised more questions than provided answers concerning the benefit-risk balance associated with these interventions. In this context, it appears important to reflect on the conditions under which a promising COVID-19-related intervention can be offered to the population.

In the field of health technology assessment, there is a consensus that recommendations regarding the population's access to promising interventions should be in full compliance with the standards and principles for demonstrating their efficacy and safety, namely the harmonized clinical practices set out by the International Council on Harmonization of Technical Requirements for Pharmaceuticals for Human Use (1). Such recommendations should also be in full compliance with the standards and principles applicable to the assessment of drugs, technologies and interventions in health and social services, including scientific rigor, equity, and the fairness and reasonableness of their use (2). Tensions surrounding the equilibrium required when applying these standards quickly emerged during the pandemic emergency, putting pressure on the social choices to be made. At the center of this situation lies the need to assess the balance between the benefits to the population (e.g., reducing strain on the healthcare system) and the risks to individuals (e.g., adverse events), in a setting of considerable uncertainty regarding the developing body of scientific evidence. It is in this context that the Institut national d'excellence en santé et en services sociaux (INESSS) conducted a reflection aiming to identify ethical considerations that could support the benefit-risk balance assessment of a promising intervention in the context of a pandemic. INESSS's mission focuses on the assessment of drugs, technologies and interventions in healthcare or social services. For this reason, “promising interventions” in this article include drug treatments and healthcare interventions provided to individuals being treated for COVID-19 disease. They do not concern vaccines or public health measures deployed to contain the pandemic.

The aforementioned reflection was initiated near the beginning of the COVID-19 pandemic, with the publication of a first “rapid response” in April 2020 regarding access to promising treatments and interventions in the pandemic context (3). This was followed by the publication, in June 2021, of a second rapid response regarding ethical considerations relevant to the assessment of the benefit-risk balance of promising interventions, entitled “Les fondements éthiques de l'évaluation de l'équilibre bénéfices-risques d'un traitement prometteur en contexte de pandémie” (4). In the present article, we push this work forward and examine the process by which the latter rapid response was produced, and the challenges raised when generating scientific evidence, evaluating promising interventions and assessing their benefit-risk balance in a pandemic context. We also explore the ethical considerations that can facilitate such an assessment, focusing on contributions to the reflection by patients and citizens. As pandemics evolve and novel pathogens and variants emerge around the globe, the need for promising interventions will continue to put pressure on the social choices to be made regarding their access, making it even more urgent to include ethical considerations stemming from various actors, including patients and citizens, in the assessment process.

In addition to considering the clinical dimension of promising interventions, the benefit-risk balance considers all the societal benefits and risks associated with the populational, sociocultural, organizational, and economic aspects regarding access to promising interventions. While presenting the major challenges identified in the scientific literature and the initiatives put forward by some regulatory agencies, this article mainly focuses on the various perspectives that were expressed in discussions that brought together ethicists, clinicians and researchers involved in clinical or public health practice, as well as patients and citizens affected by health issues related to the pandemic. It specifically aims to illustrate the crucial role played by patients and citizens as participants in the reflection and emphasizes their influence on the identification of ethical considerations aiming to address uncertainty in the assessment of the benefit-risk balance of promising interventions.

## Methods

The reflection included an initial rapid literature review that allowed exploration of the challenges identified, related to generating scientific evidence in a pandemic context and assessing the benefit-risk balance of promising interventions. This literature review served as the basis for the discussions held with the various social actors affected by the evaluation of pandemic-related interventions and decision-making on their access by the population.

### Literature Review

#### Search for Publications

For the purposes of this discussion, a strategy was developed in February 2021, in collaboration with an information specialist, to search for articles on the assessment of benefits and risks associated with promising interventions in a pandemic context, published in English or French since 2015 (Appendix A). The publication year limit was set as 2015 to cover discussions from the most recent epidemics, including those involving the Middle East respiratory syndrome coronavirus and the Ebola virus. The MEDLINE database and the Google and Google Scholar search engines were searched using keywords, which included the following: pandemic; epidemic; outbreak; benefit-risk evaluation; promising; new drug; drug use; intervention; responsibility; solidarity; justice; benefit-sharing; burden-sharing; equity; fairness; minimization of risks; maximization of benefits; unmet needs; integrity; harm reduction; beneficence; resource allocation; statistical significance; clinical significance. In October 2021, this strategy was renewed, focusing on articles published after February 2021 in order to capture the most recent literature. The websites of Health Canada, the U.S. Food and Drug Administration, and the European Medicines Agency were also searched in May 2021 to identify the main guidelines developed for evaluating promising interventions during the COVID-19 pandemic. In addition, a search for similar articles based on the studies by Califf et al. (5) and Ogburn (6) was conducted in PubMed. The search strategy yielded one pertinent reference from 2014, which was also included in the review.

#### Publication Selection

The initial search yielded 995 articles, which were examined by a single reviewer due to human resource and time constraints. The analysis of the titles and abstracts resulted in the selection of 62 articles possibly relevant to the topic of the benefits and risks associated with promising interventions in a pandemic context. The retained publications included reviews, commentaries, editorials, qualitative research and ethics articles. Documents not dealing with the benefit-risk assessment of interventions were excluded. Thirty-four articles were then read by the single reviewer. Documents concerning the analysis of the benefits and risks of a specific intervention were excluded to focus the extraction on a more general discussion concerning benefit-risk balance assessment. The second search yielded 1,677 articles from which 5 publications were selected after the application of our inclusion criteria. A total of 25 articles were included in our final literature review.

#### Data Extraction and Synthesis

Data extraction was carried out on the 25 articles by a single reviewer due to human resource and time constraints. Extraction aimed to identify the various pieces of information and positions in the literature regarding the challenges, limitations and issues associated with the benefit-risk assessment of promising interventions and its underlying considerations.

### Consultation Process

#### Group Discussions

Two group discussions were held to gain more specific insight into the experience of assessing the benefit-risk balance of promising interventions in Québec during the COVID-19 pandemic. The objectives of these group discussions were to more clearly understand the influence that the pandemic context can have on assessment activities and to provide INESSS with information about the considerations that could be proposed for assessing the benefit-risk balance of promising interventions.

The first group discussion aimed at bringing together and exchanging on the perspectives of research ethics boards, scientific evaluation committees, peer review committees that adjudicate the results of the numerous research projects underway on promising interventions, and patients and citizens directly affected by the social choices involved. Participants were selected through purposeful sampling and network sampling. Experts were recruited by personal invitation. Citizens were recruited through a call for participation to those serving on INESSS's advisory committees, in order to promote diversity of opinion on the topic. Lastly, a patient coordinator from the Methodology and Ethics Office with keen interest in ethical issues also participated in the discussion and was involved in recruiting a person who had developed COVID-19 disease in the previous year. A total of 13 people participated in a discussion held in February 2021, including ethicists, clinicians, a pharmacist, researchers, patients and citizens.

The second group discussion aimed at increasing understanding of the patient's perspective concerning the assessment of pandemic-related interventions and the conditions for their access by the population. This group discussion involved members of the Citizen Partners Committee of the Center of Excellence on Patient and Public Partnership (CEPPP), a committee made up of patient partners and caregivers who have an interest in and have taken a position on various topics pertaining to the COVID-19 pandemic. A professional scientist, a medical consultant and a patient coordinator from the Methodology and Ethics Office met with 13 members of the CEPPP committee during one of its regular meetings in March 2021.

Both meetings were recorded with the attendees' consent, and notes were taken. The notes were supplemented by the recordings. The consultations were rapidly analyzed to identify the main themes identified by the participants and the observations and positions relevant to the discussion. This analysis revealed the challenges and limitations of generating evidence and of assessing the benefit-risk balance of promising interventions encountered during the COVID-19 pandemic. It also identified some issues and considerations that might be important for the benefit-risk assessment of promising interventions.

Participants were selected for their particular interest in the topic. Conflicts of interest and roles were declared and disclosed in accordance with the *Politique de prévention, d'identification, d'évaluation et de gestion des conflits d'intérêts et de rôles des collaborateurs de l'INESSS* (Policy for the Prevention, Identification, Evaluation and Management of Conflicts of Interest and Roles of INESSS Collaborators). Nine participants from the February 2021 consultation declared having been involved in at least one committee involved in COVID-19 healthcare organization or decision-making, or an evaluation committee at INESSS before taking part in our consultation. Furthermore, 10 members of the Citizen Partners Committee reported serving on at least one committee concerned with COVID-19, such as a committee on health technology utilization, mental health, medications or the impact of COVID-19 on immunocompromised individuals, or on a health policy group associated with the Fonds de recherche du Québec. One person reported serving on a committee led by Pfizer on a topic other than COVID-19.

Participants at the February 2021 consultation served as external reviewers of the second rapid response published by INESSS to ensure that the reported perspectives accurately reflected the discussions held. The results of the two group discussions are reported herein and integrated within the current reflection, complementing the findings from the scientific literature.

## State of Knowledge and Actors' Perspectives on the Challenges in the Assessment of Promising Interventions

This section first presents the challenges of generating evidence in a pandemic context, particularly regarding the efficacy and safety of promising interventions. It then presents the challenges this situation poses for assessing their benefit-risk balance.

### Challenges of Generating Evidence in a Pandemic Context

According to the literature, only a few promising interventions were approved or recommended during previous pandemics (7). Various factors have been identified to explain this low approval rate. Most of these have to do with the context in which research is conducted during a pandemic and pose challenges for generating sound scientific evidence concerning the efficacy and safety of interventions, rendering it difficult to assess their benefit-risk balance. These challenges result from the influence that the context has on the methodological designs of the clinical trials, and from the limitations they impose on the quality of the evidence produced.

#### Influence of the Context on Methodological Design

The factors that can influence methodological design include the rapidity with which studies are conducted, time and participant recruitment constraints, and a lack of organization and coordination to allow for quick launching of pertinent research projects (8–10).

The sense of urgency and the generally short but intense duration of pandemics can explain the desire of the scientific community to promptly provide effective interventions to the population. In such a context, trials are often carried out quickly, which can lead research teams to propose methodological changes that depart from usual clinical research practices (9). Specifically, trials might be conducted without a control group and might involve the administration of concomitant interventions, which is likely to yield only a suboptimal estimate of their efficacy or safety (9). In addition, long-term trials are difficult to conduct during a pandemic, which forces research teams to adopt methodological designs that take the time constraint into account.

The emergency context can also influence the size of the cohorts included in the trials. On the one hand, the number of people who can participate in the clinical trials varies according to the course of the pandemic. For example, the ending of a pandemic can cause trials to stop before clear efficacy or safety results on the interventions are obtained (11). On the other hand, this context makes participant recruitment difficult, resulting in many studies being conducted with cohorts that are too small to obtain meaningful results representative of the clinical reality (8). The experts that took part in our consultation mentioned that the risk of over-soliciting COVID-19 disease positive individuals made participant recruitment difficult during the COVID-19 pandemic. They also stressed that some institutions imposed exclusivity with respect to specific research projects on themselves, limiting the recruitment of participants for other research projects. They argue that these challenges highlighted the need to centralize participant recruitment and to better coordinate their allocation to the various ongoing trials, at least at the organizational level.

Participant over-solicitation and the need for coordination of research projects conducted in a pandemic context at the national and international level are also identified in the literature as major issues that can influence the quality of the methodological design of clinical trials. Franks et al. showed in their study that there has been an increasing misalignment between the location of trial sites and COVID-19 geographic incidence, demonstrating the importance of coordinating pandemic research efforts (12). In view of these issues, Meyer et al. propose that a system for prioritizing research projects should be established to identify the highest-quality projects, i.e., those that permit a certain complementarity in terms of target populations and types of intervention (13). The implementation of such a system could help foster equity in the development and delivery of promising interventions for population groups in vulnerable situations in a pandemic setting (14).

The challenges associated with the course of the pandemic, participant recruitment, and research project coordination can result in changes to the methodological design of clinical trials and reduce the pool of participants available for research, rendering it difficult to obtain sufficiently clear results in a timely manner. As a solution, Dean et al. suggest using core protocols to study the use of multiple interventions for the same disease or the use of one intervention for multiple diseases simultaneously, to increase the likelihood of obtaining clear evidence (10). Others describe the importance of shared infrastructure to increase trial efficiency and reduce the threat to the scientific rigor that may arise in a context of urgency (15). Adaptive trial initiatives such as the REMAP-CAP platform and the RECOVERY and SOLIDARITY trials are excellent examples. The REMAP-CAP platform is an international initiative launched in 2019 that includes multiple sites in Europe, Australia, New Zealand and Canada and whose goal is to determine the efficacy of various interventions in reducing mortality in patients with severe community-acquired pneumonia (16), while the RECOVERY trials, a British initiative, and SOLIDARITY, a World Health Organization (WHO)-led initiative, were launched during the COVID-19 pandemic (17). Among other results, these initiatives have led to a certain level of coordination in recruitment, randomization, and trial prioritization.

#### Limitations to the Quality of the Evidence

The greatest challenges for trials conducted in a pandemic context appear, however, to have to do with demonstrating the real efficacy and safety of interventions (5). Although many publications have suggested that some of the interventions being investigated have potential benefits in treating COVID-19 disease, it has been difficult to make a clear ruling about their actual efficacy based on clinical trials involving larger cohorts (18). This situation is far from unique to the COVID-19 pandemic, having also been confirmed during the recent Ebola, Zika, and Severe acute respiratory syndrome epidemics (7).

In addition to the difficulties encountered in demonstrating the efficacy of promising interventions, it has been found that many of the COVID-19 interventions undergoing trials are accompanied by adverse effects significant enough to call their safety into question (19). In particular, uncertainty regarding the efficacy and safety of the interventions may have led regulatory agencies to recommend against their use outside of a clinical trial or to limit their use to certain situations, as the World Health Organization (WHO) and INESSS have done. Yet, it can be complicated for research teams to distinguish between adverse events that result from the course of the patient's disease and those related to the intervention (20, 21). In the context of COVID-19, the care pathway, the presence of comorbidities, and the stage of the disease all appear to be determinants of patient survival or death (21). Sex and gender also seem to influence patient mortality and individual response to the promising interventions. However, according to Brady et al. COVID-19 clinical trials have rarely taken these factors into consideration, undermining the generalizability of their results (22). In addition, safety data on promising interventions undergoing trials are sometimes missing from publications or registries, which limits their dissemination within the scientific community (2). In this context, Bhatt recommends that research ethics boards conduct an ongoing assessment of the benefit-risk balance of the different clinical trials underway (23).

The experts consulted also stressed the potential benefit of obtaining umbrella ethics approval, i.e., authorizing the conduct of multiple clinical trials for an intervention in several diseases that have similar effects on patients, such as respiratory diseases, so that trials can be launched more quickly if a pandemic emerges. In this regard, Dean et al. suggest that, despite the ending of a pandemic, it is not desirable to shut down related research projects, but rather keep them active so that they can restart quickly when an epidemic involving the same infectious agent re-emerges. To do this, they note the importance of having an independent data monitoring committee to monitor research and make recommendations relating thereto (10). Groβhennig and Koch point out that early termination of clinical trials is likely to make their evaluation by responsible organizations and agencies more challenging (24). Like Dean et al., they note the importance of relying on the recommendations of an independent data monitoring committee to support informed decision-making about shutting down projects. In the Canadian context, the need for independent monitoring committees is also mentioned in the Tri-Council Policy Statement (TCPS2). To be considered independent, this committee should normally have little or no particular interest in the research underway, the manufacturer or the research team, nor administrative responsibilities within the institution hosting the research, to prevent situations of actual, potential or perceived conflicts of interest (25).

The challenges of clearly demonstrating efficacy and identifying adverse effects attributable to the interventions can influence the quality of clinical trial evidence. This makes it very complex to assess the benefit-risk balance of the various interventions, including assessing the potential impact of introducing them into clinical practice (6).

### The Challenges of Evaluating Promising Interventions

In response to the sense of urgency that accompanies pandemics, regulatory agencies are proposing evaluation and authorization mechanisms aimed at ensuring speedier access to promising interventions by the population. Some of these mechanisms are described in INESSS's April 2020 rapid response and precede the COVID-19 pandemic. Several regulatory agencies have instituted such mechanisms during the COVID-19 pandemic or have developed specific guidelines for evaluating promising COVID-19 interventions. For example, as early as April 2020, the U.S. Food and Drug Administration (FDA) announced its emergency program, CTAP (Coronavirus Intervention Acceleration Program), for expediting the evaluation of promising COVID-19 interventions. The FDA states that it is using all available means to conduct evaluations and plans to continuously evaluate intervention data as the results from ongoing clinical trials are released. At the time the present article was submitted for publication, ~470 clinical trials had been evaluated through this program, which has resulted in 11 interventions being authorized for access through the emergency use program, and one being approved for unrestricted use in COVID-19 disease (26).

The European Medicines Agency (EMA) adopted similar initiatives to those of the FDA to support the development of promising COVID-19 interventions and accelerate their evaluation procedures (27). These initiatives stem from a plan to manage emerging health hazards that the agency adopted in 2018 (28). For its part, Health Canada adopted interim orders to expedite the approval of drugs, vaccines and medical devices related to management of COVID-19 in Canada, as well as to regulate COVID-19 drugs sale and importation (29–31). Certain key elements stemming from these interim orders have now been officialized by the adoption of the Regulations Amending Certain Regulations Concerning Drugs and Medical Devices (Shortages), published in September 2021 (32). Regulatory agencies are thus contributing to disseminating efficacy and safety data on promising interventions sent to them for the purpose of their ongoing evaluation processes.

Applying these various mechanisms and guidelines nevertheless requires an evaluation of the efficacy and safety of promising interventions, which is subject to the challenges identified in generating evidence in a pandemic context, particularly regarding benefit-risk balance assessment. In addition, the nature of the outcomes measured, the relevance of the cohorts selected in relation to the intent of the interventions, and the choice of analyses performed can all contribute to rendering this evaluation difficult.

### Challenges of Benefit-Risk Balance Assessment

Assessing the benefit-risk balance of promising interventions is a necessary step in decision-making regarding their access by the population (33). This assessment is distinct from the evaluation carried out by research ethics boards when approving the conduct of projects. Indeed, the considerations differ when going from evaluating the expected benefits and the potential risks for the participants in a controlled research setting to that of assessing the reasonably expected benefits and actual risks incurred for the population. Those responsible for making this assessment therefore must navigate through the uncertainty surrounding the evidence from clinical trials conducted during the pandemic emergency.

Reconciling the considerations concerning the acceptable benefit-risk balance for the population in general, and for individuals according to their particular situation, can prove to be exceedingly complex in the context of a health emergency, especially if the individual benefits or risks appear small while the public health benefits or risks appear significant, or vice versa (14). The media attention that sometimes accompanies intervention assessment processes and the scientific community's culture, which favors siloed scientific production, are also factors that can influence individual and social perceptions concerning the recommendations for or against access to promising interventions (8). All these factors are likely to make the benefit-risk balance assessment difficult for the evaluation team. The experts consulted reported having encountered such pressures during the evaluation of certain promising COVID-19 interventions, especially concerning their potential impact on the course of the pandemic.

During the COVID-19 pandemic emergency, many of the interventions being tested were previously approved for other disorders or diseases. The perceived advantage of testing pre-existing interventions is that they have already been shown to be safe in a clinical research setting. However, it is still important to assess all the safety parameters of interventions in the particular setting of the current pandemic, including specific responses to the infectious agent and interactions with any concomitant intervention (33). Penman et al. stressed the importance of assessing the benefit-risk balance of a given intervention before considering its use for COVID-19, especially if it is an intervention for preventing infections. In addition, Bellera et al. point out that it cannot be assumed from the prior safety demonstration of an intervention being evaluated for repurposing that it has an acceptable benefit-risk balance for the intended populations (34). Furthermore, the safety demonstration of an intervention can evolve in light of new results. Those who evaluate promising interventions can therefore face significant uncertainty regarding their benefit-risk balance. Some experts that took part in our consultations clearly expressed a preference for not granting access to an intervention when in the presence of such uncertainty.

**Patient and citizen perspectives**

Among the patients that took part in our consultations, some who felt more susceptible to the potential adverse effects of interventions for which there remains uncertainty expressed a preference for applying the precautionary principle in their personal decision-making, to avoid exposing themselves to risks. However, these positions were mitigated by those of other participants in the consultations, as the following discussion demonstrates.

Our consultations thus highlighted the importance of shared responsibility in decision-making regarding access to promising interventions. Indeed, the uncertainty stemming from the efficacy and safety data of an intervention is in tension with patients' health needs, but also with those of the general population in this context. The considerations specific to the respective responsibilities borne by the different stakeholders in this regard appear to be key elements in the discussion of the interventions' benefit-risk balance, particularly with respect to the resulting individual vs. populational responsibilities.

#### Individual Responsibility

The assessment of what constitutes an acceptable benefit-risk balance varies from one individual to another and according to the context in which the person finds herself (e.g., life stage, the presence of comorbidities, and a predisposition to risks) (33). Papadimos et al. argue that this assessment is value-laden and should, at the individual level, respect the patient's own values and priorities.

Regarding this question, Li et al. surveyed COVID-19 patients about their preference for obtaining standard care, participating in a randomized clinical trial or having immediate access to a promising intervention (35). Their results show that most of those surveyed with mild or moderate COVID-19 disease would prefer to participate in a randomized trial of a promising intervention, while those with severe disease would prefer to have direct access to the promising intervention.

**Patient and citizen perspectives**

The patients consulted for the purpose of this reflection also indicated that the form of the disease could influence their eagerness to have prompt access to a promising intervention. Most of these patients expressed their support for prioritizing knowledge building about promising interventions through research. However, they said that for some people with a severe or very severe form of the disease, it might be preferable to have access to these interventions without having to participate in a research project, even if there is no clear demonstration of an acceptable benefit-risk balance to justify such access. Similarly, these patients were of the opinion that people with a high-risk profile for developing serious complications of the disease should have the possibility of direct access to promising interventions. This would not necessarily be the case for people with few or no symptoms.
During both consultations, the patients and citizens expressed a position strongly in favor of individual responsibility for assessing the benefit-risk balance of promising interventions. In their view, this responsibility takes the form of a shared decision between clinicians and patients and the expression of the latter's free and informed consent. In this sense, they believe that it is essential to respect the patient's choice regarding the possibility of receiving a promising intervention, while ensuring that they are provided with all the information necessary for understanding the uncertainty about the benefits and risks that this might entail. The patients and citizens confirmed that the expression of a position in favor of an intervention by bodies or agencies responsible for its evaluation can increase the level of trust in these interventions. However, these persons felt that a favorable position would not prevent them from making a free and informed decision about them.

The experts consulted agreed on the importance of respecting individual patient choice for interventions that have been approved for clinical use by regulatory bodies or agencies.

#### Populational Responsibility

The preceding discussion therefore raises the question of responsibility for the benefit-risk assessment of promising interventions for the population more generally. This populational responsibility is held by various actors (e.g., researchers, manufacturers, HTA and regulatory agencies, and governmental bodies) integrity and social consciousness to ensure that the choices made concerning access to promising interventions are well-reasoned. The emergence of a pandemic creates a sense of urgency for developing interventions, in the first instance to save as many lives as possible, but also to reduce strain on the population and healthcare systems.

Thus, when there is a lack of evidence from randomized clinical trials to inform decision-making regarding promising interventions, regulatory bodies and agencies have sometimes had to rely on other types of data to make recommendations about which clinical practices to endorse (6). According to Ogburn, this may have led to opaque decision-making, which is subject to influence by political and media pressure surrounding the pandemic. The need for greater transparency in communicating the benefits and risks associated with decisions made to reduce the impact of the pandemic on the population was also raised during our consultations. To be responsible, this transparency should not be subject to such media or political pressure.

Indeed, some manufacturers and research teams conducting research on interventions previously approved for other disorders or diseases have used the media space or arenas reserved for scientific prepublication to promote the potential benefits and expected low risks of the interventions on which they work. Although this has resulted in faster sharing of research results, such information has sometimes been disseminated prematurely, which could have influenced the public's perception of an intervention's benefit-risk balance and increased pressure on the teams responsible for its assessment (19). Furthermore, a meta-analysis published by Bellos suggests that COVID-19 intervention research is susceptible to “white hat bias,” leading to greater reporting and more citations of positive vs. negative effects of promising interventions within the scientific realm (36). He argues that this type of bias may have contributed to propagating beneficial over neutral or harmful outcomes and increased the risk of creating medical misinformation concerning pandemic-related interventions of uncertain effect.

In this regard, the Council for International Organizations of Medical Sciences (CIOMS) states that in order to be considered ethical, research must have social value. In other words, it must demonstrate the relevance and reliability of the information it can generate (37). Generating information from projects with social value is considered an important step for informing access-to-intervention decision-making in an emergency context. However, prematurely disseminating information about promising interventions can influence the public's perception of their relevance and reliability, which makes informed decision-making difficult. CIOMS also mentions the risks associated with conflict between the interests of manufacturers or research teams and those of communities that access-to-intervention decisions can entail, particularly when it comes to ensuring fair and equitable allocation of limited health resources.

Buruk et al. analyzed both WHO's International Clinical Trials Registry platform and clinicaltrials.gov to verify whether the registered COVID-19 trials included information regarding various ethical criteria, including study design, conflicts of interest, enrollment of healthcare workers, and participant-related issues (38). They found that most registered studies showed inconsistencies regarding trial phases and lacked information on conflicts of interest. The effect that prematurely disseminating information can have and the risk of conflicts of interest that can emerge from research seem to be elements to consider for ensuring responsible decision-making for the population. With this in mind, the consulted experts said that in the absence of sound evidence on the efficacy and safety of a given intervention, it would be best to continue research on it. On the other hand, they noted that the issue could be viewed differently if research is not available to the population. The decision to limit access to promising interventions to the research setting until clear evidence is obtained should therefore be based on the possibility of actual access to such research.

The fact that many of the promising interventions being tested are already approved and used to treat other disorders or diseases has also had a detrimental impact on their allocation. Among other outcomes, this has led to a risk of shortages of or restricted access to some of the repurposed interventions (6, 9, 14). It therefore appears that the use of such interventions can have consequences for others in the population and thus can create an unanticipated populational risk that should be considered.

According to the experts consulted, a prioritization and coordination mechanism must be put in place to manage the supply of promising interventions once they have been approved and to reduce undesired impact on various groups. Furthermore, the approval of new interventions or the repurposing of promising ones require the assurance that the supply system has sufficient capacity to produce them, given that their use is to be recommended in the context of a pandemic. If there is no such assurance, initiating a transparent access prioritization exercise will be required, as well as proposing alternatives to the interventions concerned, if deemed necessary.

## Ethical Considerations to Support Benefit-Risk Balance Assessment

Based on our literature review and the consultations conducted for the purpose of this reflection we are able to identify considerations that may be useful to bear in mind when evaluating promising interventions. Although the benefit-risk balance is often associated with the clinical aspects of interventions, it quickly became apparent that the considerations identified concern different dimensions of the assessment process and require an assessment of their global value. These considerations are presented below while exploring the dimensions used to assess the global value of interventions at INESSS, namely, the clinical, populational, sociocultural, organizational and economic aspects (39).

### Clinical Considerations

One of the first considerations raised during the consultations was the influence that the severity of the disease can have on the pandemic emergency. It was suggested that a high mortality rate in the infected population (e.g., as with Ebola), coupled with the rapid spread of the disease, can foster the perception that the urgency of the situation justifies greater tolerance of risks or uncertainty regarding a promising intervention.

Furthermore, the clinical severity of the disease can vary, depending on the individual's profile. The characteristics that define such a profile include, among others, the form of the disease (mild, moderate or severe), the individual's overall health status (presence of comorbidities, stage of the disease, predisposition to complications), the care trajectory (pre-hospitalization, hospitalization, use of mechanical ventilation), and the intent of the promising intervention (a reduction in symptoms, in hospitalization, in the use of mechanical ventilation, or of mortality). In this regard, Penman et al. propose that it might be acceptable to expose patients with severe late-stage COVID-19 disease to a given intervention, whereas this would not be acceptable at all to patients with a mild or moderate form of the disease (33). The benefit-risk balance of access to promising interventions could thus vary according to the patient's profile. Nevertheless, the consulted experts stated that to consider access to an intervention acceptable, it cannot carry risks exceeding those that the disease itself poses.

**Patient and citizen perspectives**

The patients and citizens consulted spoke of the importance of taking into consideration an individual's willingness to accept a certain amount of risk with regards to the interventions that might be required in an emergency. This position highlights the dilemma that can arise between populational considerations in a public health emergency and individual considerations in an emergency care situation in the context of a pandemic. The differences identified in the clinical profiles that people might have can influence the perception of the benefit-risk balance of using a promising intervention for which efficacy or safety is uncertain.
The patients and citizens also indicated that some of the characteristics identified justify the idea that decisional responsibility for using a promising intervention should be borne by the individual (i.e., individual responsibility). These characteristics include, in particular, having a severe form of the disease, being hospitalized and potentially requiring the use of mechanical ventilation, having comorbidities or a predisposition to severe complications, and receiving an intervention intended to reduce the need to use mechanical ventilation or decrease mortality.

The discussion between the experts, patients and citizens allowed for the identification of the characteristics that can shift the decision regarding access to promising interventions toward a populational or organizational responsibility, such as facing a mild or moderate form of the disease, not needing hospitalization, the absence of risk factors for complications of the disease, and using an intervention intended to prevent hospitalization or reduce pre-hospitalization symptoms. Furthermore, the social context and the populational emergency that characterizes the pandemic can also influence the level of responsibility involved. It therefore seems that responsibility for these issues might reside at different levels, depending on the individual or populational priorities. Still, the responsibility for managing a pandemic lies primarily with the various policy-making bodies, such as healthcare facilities, health ministries, and regulatory bodies and agencies.

### Populational Considerations

One important consideration is the need to reduce the strain that a pandemic puts on the population and the healthcare system. It appears that regulations concerning access to promising interventions can have a negative impact on certain population groups or the healthcare system itself. This is particularly the case when one considers that few clinical trials have focused on the needs of vulnerable groups in the population, such as children or pregnant women (38). In addition, the risk of supply shortages associated with certain promising interventions for COVID-19 disease has illustrated the pressure that can occur in this regard in a pandemic context, particularly regarding the treatment of chronic diseases or other acute care situations. In response to this risk, Health Canada adopted an interim order on November 27, 2020 concerning drug shortages to safeguard the supply of medications.^1^ This order was intended, in part, to respond to the U.S. Department of Health and Human Services' September 2020 Importation of Prescription Drugs Final Rule, which was intended to facilitate the importation of interventions from Canada.

Edwards points out that overly restrictive regulations regarding the use of repurposed interventions can result in these being given or prescribed without their efficacy and safety being monitored (40). This is especially likely to occur with over-the-counter medications and can contribute significantly to creating a shortage of such drugs (9, 40).

**Patient and citizen perspectives**

In this regard, the patients and citizens we consulted agreed with the experts that prescribing or providing access to promising interventions that are used to treat other disorders or diseases requires a value judgment about the impact this practice can have at the population level.

Indeed, the shortages for certain treatments or interventions that this can cause in the population raise justice and equity issues regarding the allocation of healthcare resources (9).

### Sociocultural Considerations

The interventions of interest must demonstrate real added value to justify proposing their use. Alexander et al. point out that interventions must be proven effective and safe based on evidence from rigorous clinical trials validated by an equally rigorous peer review process (9). They believe this to be an essential condition for informed decision-making and that the circumstances of the pandemic emergency cannot transform flawed data into robust results. In May 2021, the International Coalition of Medicines Regulatory Authorities (ICMRA), an international coalition of regulatory bodies from 30 countries including Canada, reaffirmed the importance of being able to verify the integrity of clinical trials data to ensure regulatory decisions will not adversely affect patients using the medicines. For this to occur, they argue that “data must be robust, exhaustive and verifiable, through peer review” (41).

For their part, the consulted experts stated that efficacy and safety demonstrations spelling out the uncertainty associated with the interventions are required for one to be able to make informed decisions about them, both at the populational and the individual level. However, both the literature and our consultations suggest applying this precautionary principle in decision-making regarding access to interventions can also entail risks for the population, having a possible paralyzing effect on the development of promising interventions.

**Patient and citizen perspectives**

Similarly, to the experts consulted, the patients and citizens that participated in our consultations suggested that, while applying the precautionary principle is warranted in some contexts, it might be useful to remain more agile and open to revising decisions that have been made in order to permit an ongoing evaluation of an intervention's public health benefits. This evaluation may require gathering and evaluating data in real-world care settings, at least with respect to observational data that can support the benefit-risk balance assessment in care settings.

One of the experts consulted added that in such cases decision-making should remain a shared choice between clinicians and patients so as not to paralyze public health programs.

### Organizational Considerations

It seems crucial to examine the capacity of policy-making bodies and the healthcare system to deal with different levels of priorities during a pandemic emergency. Indeed, the recommendations made regarding access to promising interventions should consider the feasibility and ability of the healthcare system's actors and its organizational capacity to implement them. In this regard, decision-making should be aimed at streamlining the processes for implementing the recommendations at a time when healthcare institutions are sometimes overwhelmed by the pandemic's impact.

**Patient and citizen perspectives**

The patients and citizens consulted agreed with the experts who participated in our discussions and suggested that the unusual context of pandemics warrants considering the exceptional nature of the situation when assessing and making decisions about access to promising interventions for the population and for individuals, depending on the situation in which they find themselves.

### Economic Considerations

Pandemics can have significant impact on a population's health, the economy and the social context. The COVID-19 pandemic has shown the extent of political, social and economic decisions that are required for its management. The emergency caused by this situation is likely to increase pressure to evaluate and provide access to interventions that seem promising. However, the scientific literature mentions the potential downside of investing in research or rolling out interventions whose efficacy and safety cannot be clearly demonstrated (8, 13). It also mentions that it may be inefficient to invest in expensive interventions that do not show any benefit in terms of reducing the number of hospitalizations, ICU time, or patient mortality.

In this regard, the consulted experts pointed out that interventions can sometimes provide limited benefit to patients, reducing the length of hospital stay only to a small degree or even proving to be toxic. It is therefore not clear that the cost of certain interventions is justified by the level of benefit. However, McCaw et al. note that reducing the length of hospital stay is not an indicator of the actual benefits that promising interventions can provide, which can lead to an under- or overestimation of out-of-hospital survival (42). This decision-making therefore requires a global assessment of the economic issues at play in a given situation. The sharing of the budgetary burden between the levels of government (national, provincial and municipal) and between the various stakeholders stands out as one of the economic issues particularly important to discuss in a pandemic context.

## Applying Ethical Considerations to the Benefit-Risk Balance Assessment

To facilitate the benefit-risk balance assessment of promising interventions, it might be useful to draw on existing models of decision-making regarding access to care or to interventions in a context of uncertainty or limited resources or in rare situations. The McGill University Health Center has developed a model that proposes integrating casuistic considerations, i.e., those rooted in a conceptualization of specific cases or contexts, into an organizational decision-making process aimed at making fair and reasonable decisions based on distributive justice considerations [(43), personal communication]. Such a model can be used to assess the benefit-risk balance by considering the above-mentioned characteristics of personal profiles, but also the populational context and the potential impact of intervention access, the priorities that emerge regarding the interventions, and the organizational capacity to manage the conditions of access and the related economic issues.

**Figure 1:**
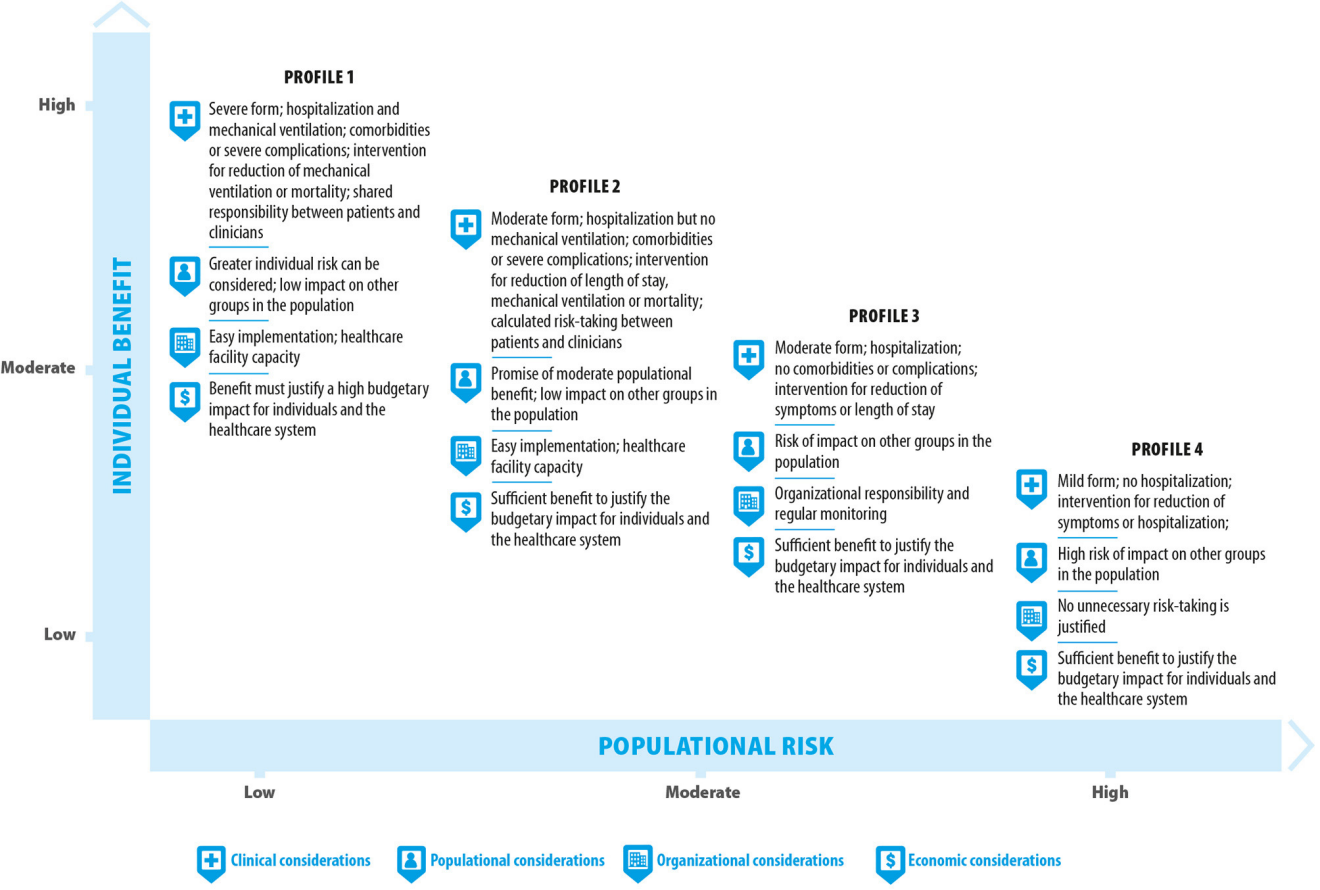
Benefit-risk assessment profiles for the evaluation of promising interventions in a pandemic context.

The benefit-risk balance could thus be described as a variable that depends on the combination of the considerations that have been identified. The integration of the perspectives from the various social actors consulted within this present reflection allowed for better understanding of the ethical considerations that can help address the uncertainty surrounding promising interventions and the proposal of an assessment approach that is sensitive to these considerations. In its 2021 rapid response, INESSS presented four situational profiles for the purpose of supporting benefit-risk balance assessments using the identified considerations (Figure 1). It should be noted that these profiles are not intended to describe all the possible combinations of the identified characteristics and considerations, but rather to provide a general framework to support the teams responsible for assessing the benefit-risk balance of promising interventions.

### Profile 1 Can Occur in a Situation Where Individual Benefit and Risk Are Expected to Be High and Populational Risk Low

This profile can occur when facing individuals with a severe form of the disease, requiring hospitalization and the use of mechanical ventilation, who have comorbidities or a predisposition to severe complications, or for whom the intervention considered is intended to reduce the use of mechanical ventilation or mortality. Based on the consultations, this profile would allow considering greater individual risk taking despite the uncertainty regarding the interventions' efficacy and safety. The decision-making process concerning access to such interventions could rest on the individual patient concerned and engage the shared responsibility of the clinician in a free and informed consent process. However, to be acceptable from a populational standpoint, access to the promising interventions should not have an adverse impact on the rest of the population (e.g., a shortage that could cause significant harm to other patients). Such access should be easily implementable in the healthcare setting and show sufficient benefit relative to its budgetary impact on the healthcare system or on individuals. In such a case, populational, organizational and economic impact should be among the considerations taken into account by regulatory agencies before allowing individual decisions to be made.

### Profile 2 Can Occur in a Situation Where Populational Benefit Is Expected to Be Moderate and Populational Risk Low to Moderate

This profile can occur when facing individuals with a moderate form of the disease, requiring hospitalization without the use of mechanical ventilation, who have comorbidities or a predisposition to severe complications, and for whom the intervention considered is intended to reduce the length of hospital stay, the use of mechanical ventilation or mortality. Given the clinical profile of those who might benefit from the interventions under such a scenario, this profile can be considered to hold promise of a moderate populational benefit. This profile might justify calculated risk taking by patients and clinicians. The decision-making process concerning access to the interventions under this scenario could rest more on shared responsibility between clinicians and patients and requires a situationally proportionate assessment of the benefits and risks as part of the free and informed consent process. However, to be acceptable from a populational standpoint, access to promising interventions should not have an adverse impact on the rest of the population (e.g., a shortage that could cause harm to other patients). Such access should be easily implementable in the care setting and show sufficient benefit relative to its budgetary impact on the healthcare system or on individuals. In such a case, populational, organizational and economic impacts should be among the considerations taken into account by regulatory agencies before allowing shared decision-making between clinicians and patients.

### Profile 3 Can Occur in a Situation Where Individual Benefit Is Expected to Be Moderate and Populational Risk Moderate to High

This profile can occur when facing individuals with a moderate form of the disease, requiring hospitalization, who have no comorbidities or predisposition to severe complications, and for whom the intervention considered is intended to reduce symptoms or the length of hospital stay. Furthermore, access to the intervention concerned is likely to create a shortage for other groups in the population. This profile does not justify taking risks concerning the uncertainty associated with the efficacy and safety of the intervention, that could exceed the risks posed by the disease to the patients concerned or to the population. The decision-making process concerning access to the interventions under this scenario lies more with policy-making and organizational bodies. This profile could require organizational monitoring to revise the benefit-risk balance assessment in light of the course of the disease and patient care trajectories. To be acceptable, this access should show sufficient benefit relative to its budgetary impact on the healthcare system or on individuals.

### Profile 4 Can Occur in a Situation Where Individual Benefit Is Expected to Be Low and Populational Risk High

This profile can occur when facing individuals with a mild form of the disease, not requiring hospitalization, and for whom the intervention considered is intended to reduce the symptoms of the disease or risk of hospitalization. Furthermore, access to the intervention in question is highly likely to create a shortage for other groups in the population. This profile engages populational responsibility on the part of policy-making bodies involved in the decision-making process. The benefit-risk assessment under this scenario should ensure that no unnecessary risks are incurred for the population, such as the risk of an intervention shortage or of unsuspected adverse effects. This access should demonstrate sufficient benefit relative to its economic impact on the healthcare system or on individuals.

Edwards defines such an approach as being adapted to the level of risk, and supports the notion that it is acceptable for the bodies and agencies responsible for evaluating promising interventions to require a lower level of evidence of benefit, in order to promote research and development of interventions for people with greater need in the context of the disease (40).

An approach adapted to the level of risk and the needs of individuals could permit differential value judgments based on their vulnerability and ensure respect of their right to try interventions, as was raised during the consultations.

## Discussion

Taking the identified ethical considerations into account suggests that the benefit-risk balance of promising interventions can vary according to the specific context of a pandemic and those most susceptible to its impact. This makes the evaluation and decision-making processes concerning promising interventions even more difficult when the evidence demonstrating their efficacy and/or safety is marked by uncertainty.

Overall, the present reflection demonstrates how decision-making concerning access to promising interventions in a pandemic context requires humility in the face of the available knowledge and the promotion of continued data collection to inform the social choices that will likely have to be made. It also suggests that the dissemination of scientific knowledge should preferably occur following its validation by peers. If deemed useful to occur prior to such validation, such dissemination should report its limitations in a clear and transparent manner. In light of this reflection, it appears that the benefit-risk balance assessment of promising interventions should take various factors into account, including:

- the severity of the disease;
- people's vulnerability to the disease;
- the uncertainty associated with the interventions' efficacy and safety;
- the populational impact of access to the interventions (e.g., risk of shortages);
- the individual and populational priorities regarding the interventions;
- the organizational capacity and feasibility of applying the decisions made; and
- the economic issues associated with access to the promising interventions.

The assessment model proposed by the McGill University Health Center for making decisions about access to care or interventions in a context of uncertainty or limited resources, or in rare situations, provides a new way of thinking about the issue of assessing the benefit-risk balance of promising interventions. The profiles proposed for conducting such assessments also appear to be supported by the risk-adapted approach described by Edwards for addressing the challenges of evaluating promising interventions in a pandemic context (40). In light of our reflection, it also seems necessary to adopt a framework involving several aspects to permit a thorough benefit-risk balance assessment and a global evaluation of promising interventions in a pandemic context. An assessment of the global value of the interventions using the model proposed by INESSS (39) would make it possible to consider all the aspects affected by the responses to a pandemic relating to the interventions being evaluated. The considerations and the approach to assessing the benefit-risk balance that emerge from this reflection can be applied to other contexts susceptible to fostering significant uncertainty surrounding the available scientific evidence, such as an epidemic setting.

While not limited to the COVID-19 pandemic, the literature review and the consultations that were carried out for the purposes of this reflection mainly paint a picture of the situation as experienced during this setting. However, the lived reality of the COVID-19 pandemic has shown the degree to which knowledge about the present subject was lacking. It seems that the lessons learned during previous pandemics were not sufficient to enable approaching the current one with confidence. Since the context in which each pandemic takes place might differ, the present discussion has limitations in terms of identifying the particular challenges that another pandemic might bring, particularly with respect to generating knowledge and assessing the benefit-risk balance of its specific promising interventions.

Regardless of the approach chosen to assess the benefit-risk balance of promising interventions, the primary responsibility for doing so still rests with the research teams and manufacturers conducting clinical trials. In this regard, clinical research conducted in a pandemic context should adhere to the standards and principles of responsible generation and dissemination of scientific knowledge, and:

- allow a clear demonstration of individual or populational benefits, taking account of the interventions' efficacy and effectiveness as well as associated uncertainty;
- report the uncertainty regarding the interventions' safety in a transparent manner;
- disseminate the research results in a timely manner;
- avoid being influenced by the urgency of the context and its accompanying pressures; and
- consider the special needs of people in vulnerable situations (e.g., pregnant women, the elderly, and people with chronic conditions and children).

Lastly, the benefit-risk balance assessment of promising interventions should seek to respect the principles of justice, equity, solidarity and transparency, which are essential for enabling the population to make free and informed decisions about their resulting supply.

## Conclusion

The consultations conducted during this reflection demonstrated how decision-making in this regard should consider both the individual and populational priorities arising from the pandemic as much as possible. The dynamic between the various social actors brought together to discuss these issues allowed us to create a space of mutual understanding of the diverse perspectives presented. As the discussion moved forward, these perspectives became intertwined and allowed for the identification of ethical considerations which respect and integrate the views of all the participants. Although these results reflect the perspectives of a limited number of individuals, it was particularly rewarding to witness how the patient and citizen perspectives contributed to a shift in the thinking about the benefit-risk balance assessment of promising interventions.

## Author Contributions

CO contributed as the lead author to writing the manuscript and conducted the literature review and the consultations. IG and MG initially suggested carrying out this reflection and contributed to the group discussions. OD-P, LL, and M-PP contributed to the group discussions. SP contributed to the English version of the manuscript. All authors commented and approved the final manuscript.

## Funding

M-PP holds the Chair of Evaluation of State-of-the-Art Technology and Methods. Citizen and Patient Engagement in the Transformation of Organizations and Health Systems at the Center hospitalier de l'Université de Montréal, jointly financed by le Fonds de recherche du Québec-Santé, theministére de la Santé et des Services sociaux du Québec and the Center hospitalier de l'Université de Montréal.

## Conflict of Interest

The authors declare that the research was conducted in the absence of any commercial or financial relationships that could be construed as a potential conflict of interest.

## Publisher's Note

All claims expressed in this article are solely those of the authors and do not necessarily represent those of their affiliated organizations, or those of the publisher, the editors and the reviewers. Any product that may be evaluated in this article, or claim that may be made by its manufacturer, is not guaranteed or endorsed by the publisher.

## Footnotes

^1^The Interim Order Respecting Drug Shortages (Safeguarding the Drug Supply). Available online at: https://www.canada.ca/en/health-canada/services/drugs-health-products/compliance-enforcement/importation-exportation/interim-order-drug-shortages-protecting-supply/guidance.html (accessed May 26, 2021).
