# Supplementary material for: Patient and Citizen Participation in the Identification of Ethical Considerations Aiming to Address Uncertainty in the Evaluation of Promising Interventions in a Pandemic Context
Source: Front Med Technol. 2021 Dec 24;3:794003. doi: 10.3389/fmedt.2021.794003 (PMC8757856; doi:10.3389/fmedt.2021.794003)
Supplement: Supplementary file 1 [file Data_Sheet_1.pdf]

APPENDIX A
Scientific information search strategy

Bibliographic databases

**MEDLINE (Ovid)**<br>**Date of search : February 2021**<br>**Limitations : 2015- ; english, french**	
1	COVID-19/ OR Coronavirus Infections/ OR SARS-Cov-2/ OR (COVID-19* OR COVID19* OR 2019-nCoV* or 2019nCov* OR coronavirus disease-19 OR ((coronavirus* or corona virus* or CoV or ncov*) ADJ2 (new OR novel OR wuhan OR china OR chinese OR 2019)) OR SARS corona virus 2 or SARS coronavirus 2 or SARS-CoV-2* OR SARS-CoV2* OR SARSCoV2* OR SARS2* OR ("severe acute respiratory syndrome" AND (corona virus 2 OR coronavirus 2 OR CoV2)) OR coronavirus* or corona virus* or CoV or CoVs OR "WN-CoV" or SARS* or "severe acute respiratory syndrome" or MERS* or (middle east* AND respiratory) OR WN-CoV OR Ebola* OR H1N1* OR avian influenza* OR H5N1* OR outbreak* OR pandem* OR epidemic* OR epidemy OR epidemis OR disaster* OR health crisis OR catastrophe* OR sanitary crisis OR sanitary emergenc*).ti,ab
2	(intervention* OR molecule* OR treatment* OR drug OR drugs OR technolog* OR product OR therap*).ti,ab
3	(trial* OR rct OR rcts).ti,ab
4	(evaluat* OR assess* OR apprais* OR interpret* OR reporting).ti,ab
5	(effect* OR safety OR safe OR safely OR (benefit* ADJ2 risk*) OR harm*).ti,ab
6	1 AND 2 AND 3 AND 4 AND 5
7	expectation*.ti,ab
8	1 AND 3 AND 7
9	6 OR 8
